# Plasma PCSK9 levels are significantly modified by statins and fibrates in humans

**DOI:** 10.1186/1476-511X-7-22

**Published:** 2008-06-11

**Authors:** Janice Mayne, Thilina Dewpura, Angela Raymond, Marion Cousins, Anna Chaplin, Karen A Lahey, Stephen A LaHaye, Majambu Mbikay, Teik Chye Ooi, Michel Chrétien

**Affiliations:** 1 Chronic Disease Program, Ottawa Health Research Institute, The Ottawa Hospital, University of Ottawa, Ottawa, Ontario, Canada; 2 Clinical Research Laboratory, Division of Endocrinology and Metabolism, Department of Medicine, The Ottawa Hospital, University of Ottawa, Ontario, Canada; 3 Vascular Disease Prevention and Research Centre for Southeastern Ontario, Queen's University, Kingston, Ontario, Canada

## Abstract

**Background:**

Proprotein convertase subtilisin kexin-like 9 (PCSK9) is a secreted glycoprotein that is transcriptionally regulated by cholesterol status. It modulates levels of circulating low density lipoprotein cholesterol (LDLC) by negatively regulating low density lipoprotein receptor (LDLR) levels. PCSK9 variants that result in 'gain of function' have been linked to autosomal dominant hypercholesterolemia, while significant protection from coronary artery disease has been documented in individuals who carry 'loss of function' PCSK9 variants. PCSK9 circulates in human plasma, and we previously reported that plasma PCSK9 is positively correlated with total cholesterol and LDLC in men.

**Results:**

Herein, we report the effects of two lipid-modulating therapies, namely statins and fibrates, on PCSK9 plasma levels in human subjects. We also document their effects on endogenous PCSK9 and LDLR expression in a human hepatocyte cell line, HepG2, using immunoprecipitation and immunoblot analyses. Changes in plasma PCSK9 following fenofibrate or gemfibrozil treatments (fibric acid derivatives) were inversely correlated with changes in LDLC levels (r = -0.558, p = 0.013). Atorvastatin administration (HMGCoA reductase inhibitor) significantly increased plasma PCSK9 (7.40%, p = 0.033) and these changes were inversely correlated with changes in LDLC levels (r = -0.393, p = 0.012). Immunoblot analyses of endogenous PCSK9 and LDLR expression by HepG2 cells in response to statins and fibrates showed that LDLR is more upregulated than PCSK9 by simvastatin (2.6× vs 1.5×, respectively at 10 μM), while fenofibrate did not induce changes in either.

**Conclusion:**

These results suggest that *in vivo *(1) statins directly increase PCSK9 expression while (2) fibrates affect PCSK9 expression indirectly through its modulation of cholesterol levels and (3) that these therapies could be improved by combination with a PCSK9 inhibitor, constituting a novel hypercholesterolemic therapy, since PCSK9 was significantly upregulated by both treatments.

## Background

*Proprotein convertase subtilisin/kexin-like *9 (*PCSK9*) [1] is a highly polymorphic gene with over 40 non-synonymous, exonic single nucleotide polymorphisms (SNPs) reported in humans [2]. Three of these PCSK9 variants cause autosomal dominant hypercholesterolemia (ADH) [3-6] while 11 others associate with a hypercholesterolemic phenotype [2]. In addition, two nonsense and several missense PCSK9 variants were reported that associated with hypocholesterolemia [7-9]. Thus, PCSK9 variants resulting in a 'gain-of-function' predispose carriers to hypercholesterolemia, whereas PCSK9 variants resulting in a 'loss-of-function' associate with hypocholesterolemia. Studies that have shown decreased risk of coronary artery disease for 'loss-of-function' PCSK9 carriers have generated great interest in the development of PCSK9 inhibitors that could improve current dyslipidemic therapies [7,10,11].

*PCSK9 *transcription is upregulated by the cholesterogenic pathway through sterol regulatory element binding protein-2 (SREBP-2) [12-14], a transcription factor important for the regulation of genes involved in cholesterol biosynthesis as well as low density lipoprotein receptor (LDLR) synthesis. PCSK9 is most highly expressed in the liver and small intestine, and is found in circulation [15-17]. Secreted PCSK9 can interact with cell surface LDLR and enter the endocytic recycling pathway, shifting the equilibrium of LDLR recycling toward LDLR lysosomal-dependent degradation, thereby modifying circulating low density lipoprotein cholesterol (LDLC) levels [16,18-22].

Recently we reported that plasma PCSK9 levels correlated to total cholesterol (TC) and LDLC in men but not in women, and that plasma PCSK9 levels may be an indicator of carriers of PCSK9 variants [17]. Since PCSK9 is a target for drug design, to improve current dyslipidemic therapies, it is important to understand how current treatments affect PCSK9 expression. We report here on the effects of two classes of lipid-lowering drugs (fibrates and statins) on plasma PCSK9 levels and their effect on PCSK9 and LDLR expression in a hepatic cell line. Collectively, our results show that: (1) plasma and secreted PCSK9 levels reflect intracellular levels of PCSK9, (2) changes in LDLC are associated with changes in circulating levels of this enzyme and (3) by monitoring plasma PCSK9 changes during dyslipidemic therapies one can identify those individuals that would benefit most from combination therapy with PCSK9 inhibitors.

## Results

### The level of fasting PCSK9 in human plasma is affected by fibrate therapy

**Figure 1:**
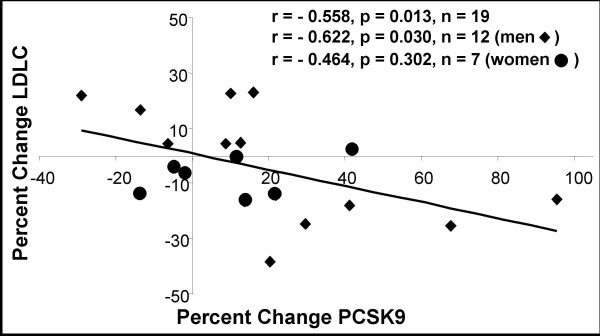
**Relationship between percent change in plasma PCSK9 and LDL-cholesterol levels following fibrate treatment**. PCSK9 and LDL-C were measured pre- and post-fibrate therapy as described in Methods. Pre-treatment levels were set as 100%.

Fibrates primarily reduce triglycerides (TG) and increase high density lipoprotein cholesterol (HDLC) levels with variable effects on LDLC [23]. Using samples from a study that compared the efficacy of two standard fibrate therapies [24], we measured plasma PCSK9 levels from 19 individuals (12 men and 7 women) pre- and post-fibrate treatment. We did not observe any significant difference in pre-treatment lipids between the fenofibrate (n = 9) and gemfibrozil (n = 10) groups or in lipoprotein parameters in response to treatments between the two groups (data not shown). Therefore these subjects were combined and further analyzed as the fibrate group (n = 19; Table 1). The average pre- and post-treatment levels, as well as percent change in PCSK9, TC, LDLC, TG, HDLC and the TC/HDLC ratio are shown in Table 1. Total cholesterol, TG and the TC/HDLC ratio significantly decreased following fibrate therapy (-11.3%; p = 0.002, -43.6%; p < 0.0001 and -22.0%; p < 0.0003, respectively), while plasma PCSK9 and HDLC increased significantly (17.0%; p = 0.031 and 15.7%; p = 0.002 respectively). Although LDLC did not show a significant change with therapy, it should be noted that 8 individuals showed an increase while 11 individuals showed a decrease in LDLC. We went on to examine the relationship between the percent change in PCSK9 and lipoprotein parameters pre- and post-therapy using Spearman's correlation. There was a significant inverse correlation between changes in the levels of PCSK9 and LDLC (Fig 1; r = -0.558, p = 0.013, n = 19), attributable to the significant association between these two parameters in men (r = -0.622, p = 0.031, n = 12).

**Table 1:** Fasting PCSK9 and lipid levels pre- and post-fibrate treatment

	Baseline	Treatment	% Change
PCSK9 (μg/mL)	6.04 ± 1.10	6.90 ± 1.38*	17.01 ± 29.5
Total cholesterol	6.22 ± 0.73	5.50 ± 0.88**	-11.3 ± 11.6
Triglycerides	3.27 ± 0.59	1.85 ± 0.80***	-43.6 ± 22.8
HDL cholesterol	0.86 ± 0.13	1.00 ± 0.21**	15.7 ± 18.4
LDL cholesterol	3.85 ± 0.65	3.65 ± 0.71	-4.22 ± 17.6
TC/HDLC Ratio	7.35 ± 1.22	5.79 ± 1.68***	-22.0 ± 13.5

Significance comparing baseline and treatment by Wilcoxon's signed rank test (n = 19). Values expressed as μmol/L except where indicated.

*p < 0.05, **p < 0.001, ***p < 0.0001

### The level of PCSK9 and LDLR in the HepG2 cell line is unaffected by fibrates

**Figure 2:**
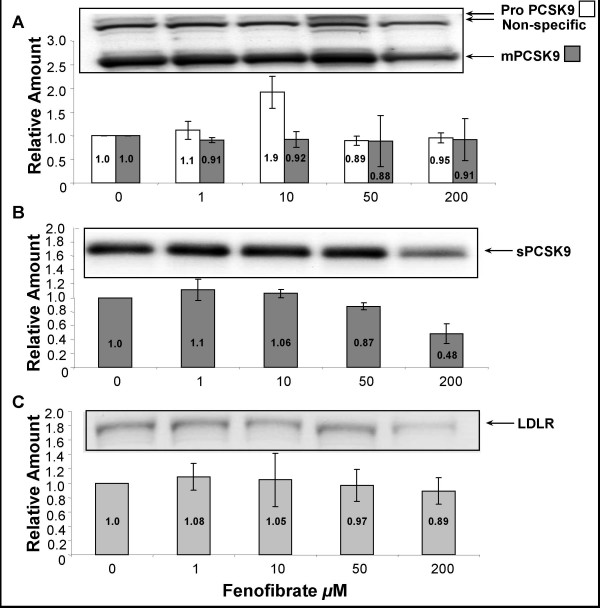
**Effect of increasing concentrations of fenofibrate on PCSK9 and LDLR protein expression in HepG2 cells**. Cells were grown in DMEM + 10% FBS and supplemented with increasing concentrations of fenofibrate for 24 hours at 37°C. 50 μg of total cell lysates (A and C) and 100 μl cell media (B) were analyzed by immunoblotting (IB), or immunoprecipitation (IP) followed by IB, respectively, as described in Methods. A and B were IB with anti-hPCSK9 Ab and C with anti-hLDLR Ab. The protein signals were quantified by densitometry using Syngene Chemigenius 2XE imager and Gene Tools software. All values were made relative to values from untreated cells set as 1 and presented as mean ± SEM (n = 3).

Figure 2 represents the regulation of the intracellular proPCSK9 and intracellular mature PCSK9 (mPCSK9), secreted PCSK9 (sPCSK9) and the LDLR (A to C, respectively), in response to increasing concentrations of fenofibrate (0–200 μM). The amount of proPCSK9 and mPCSK9 (panel A) did not change significantly, nor did sPCSK9 (panel B). As well, there was no significant response in LDLR expression over these concentrations of fenofibrate (panel C). These data indicate that fenofibrate treatment does not directly affect PCSK9 or LDLR expression in HepG2 cells.

### The level of fasting PCSK9 in human plasma is affected by statin therapy

**Figure 3:**
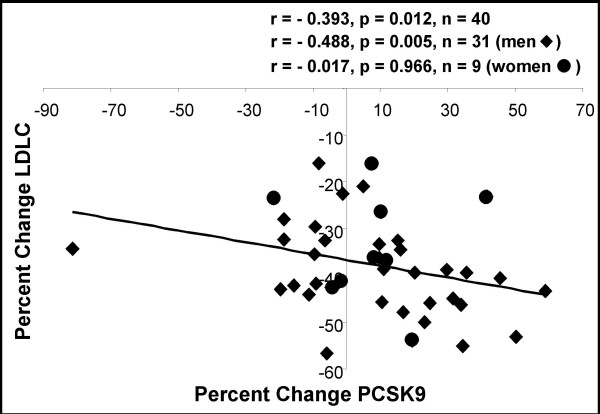
**Relationship between percent change in plasma PCSK9 and LDL-cholesterol levels following statin treatment**. PCSK9 and LDL-C were measured pre- and post-statin therapy as described in Methods. Pre-treatment levels were set as 100%.

Statin therapy lowers LDLC levels by inhibiting hydroxy-3-methylglutaryl-coenzyme A reductase (HMGCoAR), abating intracellular cholesterol levels while increasing the expression of the LDLR [25]. This serves to augment clearance of circulating LDLC through increased liver uptake [25]. To examine the effect of statin therapy on PCSK9, we measured its level from the serum of 40 individuals from a study comparing lipid parameters pre- and post-atorvastatin treatment. The average pre- and post-treatment levels, and percent change for PCSK9, TC, LDLC, TG, HDLC and the TC/HDLC ratio are shown in Table 2. Atorvastatin significantly reduced TC, LDLC and TG levels (-25.0%; p < 0.0001, -38.0%; p < 0.0001 and -16.2%; p < 0.001, respectively) while significantly increasing HDLC levels (8.03%; p < 0.0001). Plasma PCSK9 levels significantly increased by 7.40% (p = 0.035). Changes in plasma PCSK9 levels following atorvastatin therapy showed a significant inverse relationship with changes in LDLC (Fig 3; r = -0.393, p = 0.012, n = 40), which was attributable to the strong association between these two parameters in men (r = -0.488, p = 0.005, n = 31).

**Table 2:** Fasting PCSK9 and lipid levels pre- and post-atorvastatin treatment

	Baseline	Treatment	% Change
PCSK9 (μg/mL)	5.88 ± 1.70	6.32 ± 2.29*	7.40 ± 25.3
Total cholesterol	5.82 ± 0.76	4.37 ± 0.62***	-25.0 ± 9.06
Triglycerides	1.50 ± 0.56	1.26 ± 0.58**	-16.2 ± 28.0
HDL cholesterol	1.32 ± 0.26	1.43 ± 0.30***	8.03 ± 13.2
LDL cholesterol	3.82 ± 0.67	2.37 ± 0.53***	-38.0 ± 10.3
TC/HDLC Ratio	4.53 ± 0.89	3.17 ± 0.63***	-30.1 ± 9.79

Significance comparing baseline and treatment by Wilcoxon's signed rank test (n = 40). Values expressed as μmol/L except where indicated.

*p < 0.05, **p < 0.001, ***p < 0.0001

### The levels of PCSK9 and LDLR in the HepG2 cell line are affected by statin therapy

**Figure 4:**
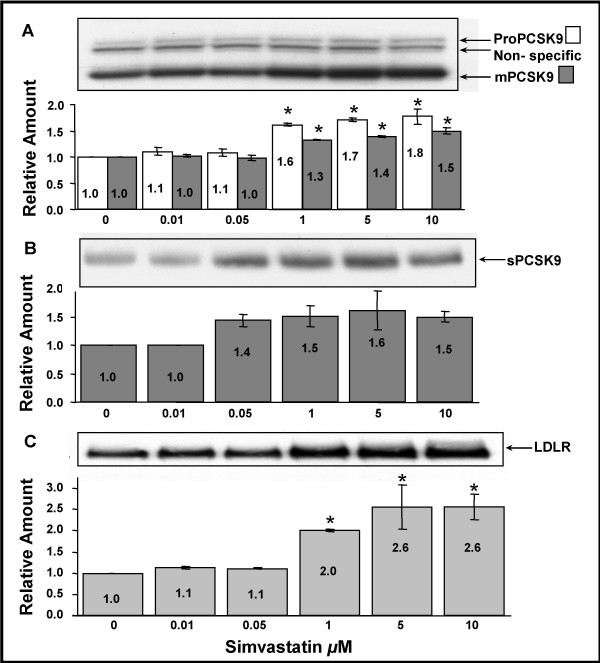
**Effect of increasing concentrations of simvastatin on PCSK9 and LDLR expression in HepG2 cells**. HepG2 cells were grown in DMEM + 10% FBS and supplemented with increasing concentrations of simvastatin for 24 hours at 37°C. 50 μg of total cell lysates (A and C) and 100 μl cell media (B) were analyzed by immunoblotting (IB), or immunoprecipitation (IP) followed by IB, respectively, as described in Methods. A and B were IB with anti-hPCSK9 Ab and C with anti-hLDLR Ab. The protein signals were quantified by densitometry using Syngene Chemigenius 2XE imager and Gene Tools software. All values were made relative to values from untreated cells set as 1 and presented as mean ± SEM (n = 3). * Indicates significant differences from untreated control (p < 0.05) using student's T-test.

Figure 4 represents the upregulation, at the protein level, of the intracellular and secreted forms of PCSK9 (A and B, respectively) and the LDLR (C) in response to increasing concentrations of simvastatin (0–10 μM). Significant upregulation of proPCSK9 as well as mPCSK9 was observed at 1 μM simvastatin when compared to untreated control cells (panel A). The same trend was observed with sPCSK9 although this did not reach significance (panel B). The ratio of proPCSK9 to mPCSK9 did not change significantly upon upregulation. As well, upregulation of intracellular PCSK9 was reflected by changes in secreted PCSK9 measurements (compare panels A and B). The LDLR was also significantly upregulated by simvastatin at 1 μM, showing a 2× increase compared to untreated control cells (panel C). From 1–10 μM simvastatin, LDLR expression was more highly upregulated than intracellular and secreted PCSK9; compare 2.6× to 1.5× and 1.5×, respectively (Fig 4).

## Discussion

In previous reports we showed that PCSK9 is efficiently secreted from HepG2 cells [1]. Herein, we show that changes in secreted PCSK9 reflect changes in intracellular PCSK9 expression in a HepG2 cell model (Figs 2 and 4). This direct link between maturation of proPCSK9 to mPCSK9 and its secretion further suggests, along with our published association of 'loss of function' PCSK9 variants with reduced levels of plasma PCSK9 [17], that plasma levels of PCSK9 can serve as a measure of regulation of this physiologically relevant PCSK in humans.

This study demonstrates that plasma PCSK9 levels are significantly modified by two common lipid therapies; fibrates and statins (Tables 1 and 2 and Figs 1 and 3). The effect of fibrate therapy on plasma PCSK9 levels has not been previously reported. Fibrates (peroxisome proliferator activated receptor alpha (PPARα) agonists) are a class of drug that is used to primarily treat hypertriglyceridemia and low HDLC. They induce the PPARα pathway and upregulate genes that encode proteins that increase HDL production and secretion, while reducing very low-density lipoprotein (VLDL) triglyceride secretion [26]. Fibrates have variable effects on circulating LDLC levels through an unknown pathway [26]. Indeed in our cohort of patients (n = 19); 8 individuals showed an increase, while 11 individuals showed a decrease in LDLC levels following fibrate therapy (Fig 1). Likewise, the levels of plasma PCSK9 both increased and decreased following fibrate treatment; overall, however there was a significant increase in plasma PCSK9 levels of 17% (Table 1 and Fig 1). As well, changes in plasma PCSK9 were significantly inversely correlated to changes observed in LDLC following fibrate therapy (Fig 1).

Our cell culture data indicated that fenofibrate (up to 200 μM) does not significantly change the expression of PCSK9 or the LDLR (Fig 2). A recent report suggests that *PCSK9 *mRNA expression can be significantly repressed in HepG2 cells by fenofibrate at concentrations >200 μM [27]. Similar to this study we also see non-significant downregulation of PCSK9 protein levels at 200 μM but not at lower concentrations. It is important to note that based on the pharmacokinetics for gemfibrozil [28] and fenofibrate [29] in humans, the subjects in our study received 100 μM and 30 μM, respectively, well below that reported to repress *PCSK9 *transcription *ex vivo*. Taken together with our data from human studies (Fig 1), it is likely that therapeutic doses of fibrates exert their effect on plasma levels of PCSK9 indirectly, in response to changes in cholesterol levels. We suggest that in the case of increased levels of circulating LDLC, intracellular levels of sterols increase, downregulating genes activated by the SREBP pathway (including *PCSK9*) and *vice versa*.

*PCSK9 *is upregulated by cellular cholesterol depletion through the sterol regulatory element binding protein-2 (SREBP-2) pathway [14]. The SREBP family includes three transcription factors, SREBP-1a, -1c and -2. SREBP-2 preferentially activates genes involved in cholesterol biosynthesis and metabolism, such as HMGCoA synthase and HMGCoAR. SREBPs -1a and -1c preferentially activate genes involved in fatty acid biosynthesis, such as acetyl CoA carboxylase and ATP citrate lyase [30]. The role of SREBP-2 in *PCSK9 *transcriptional regulation is well documented [12-14,31] while several reports suggest that *PCSK9 *is also regulated by SREBP-1c [12,13,32]. Statins inhibit HMGCoAR, a rate-limiting enzyme in cholesterol biosynthesis and have been shown to significantly increase PCSK9 mRNA in HepG2 cells and primary human hepatocytes through activation of the SREBP-2 pathway [14].

In this report, we measured plasma PCSK9 levels in response to low-dose atorvastatin therapy (10 mg/day) and show an average increase of 7.4% in circulating levels of PCSK9 in individuals following treatment (effective dosage 0.0217 μM). Another group showed that 40 mg/day atorvastatin further increased plasma PCSK9 by 34% [33]. However, they did not report the significant inverse correlation between percent change in LDLC and PCSK9 that we observed (Fig 3). This may be because their study group was smaller (n = 12 versus n = 40). The results we report here for protein upregulation of PCSK9 by simvastatin in HepG2 cells (Fig 4; 40% at 0.05 μM) was comparable to that observed in their study undergoing atorvastatin therapy (34% at 0.08 μM). Collectively, our cell culture and human studies suggest that in response to statins, increases in *PCSK9 *mRNA levels are reflected in circulating levels of PCSK9 (Fig 3).

Statins reduce LDLC levels through a second mechanism; they increase LDLR levels through the SREBP-2 pathway, further decreasing circulating LDLC. PCSK9, which can degrade the LDLR, is also upregulated by statins *in vivo *(Fig 3) and *ex vivo *(Fig 4). When we measured the effect of simvastatin on the LDLR protein expression in the HepG2 cell line, it showed significant upregulation at 1 μM (Fig 4). However, LDLR expression was more highly upregulated than that of PCSK9 (2.0× versus 1.5× at 1 μM and 2.6× versus 1.5× at 10 μM simvastatin, respectively). This suggests that although PCSK9 is a negative regulator of the LDLR and, in the case of statins, is upregulated in concert with the LDLR by the SREBP-2 pathway, the increase in PCSK9 at the protein level is not sufficient to completely negate upregulation of the LDLR.

In our study the average increase in plasma PCSK9 was greater following fibrate therapy (17%) than following statin therapy (7%). These two post-hoc study groups are exclusive in terms of participants; therefore, we cannot directly compare differences in responses between the two therapies. However, we do know that an increase in PCSK9 may limit the efficacy of therapies in terms of lowering circulating LDLC levels.

In both fibrate and statin cohorts, the inverse correlation between changes in plasma PCSK9 and LDLC was significant in men, and not in women, although the numbers of women were low and therefore may not have been significantly powered. Previously we reported a gender dichotomy in the direct relationship between LDLC levels and plasma PCSK9 in men and not women [17], suggesting that gender-specific hormones such as estrogens and/or testosterone may also affect PCSK9 transcription and/or expression. This is an avenue we are pursuing.

## Conclusion

This study demonstrates that plasma PCSK9 can be used as an accessible and meaningful measurement of its intracellular regulation. This provides useful information, such as identifying those individuals that would benefit most from combination therapy with PCSK9 inhibitors (now being developed) that could improve current dyslipidemic therapies. In conclusion, since PCSK9 was significantly upregulated by both fibrate and statin therapies, our results indicate that individuals undergoing either treatment (even at low doses) may benefit by combination with PCSK9 inhibitors, increasing their LDLC lowering effect.

## Methods

### Antibodies

The anti-PCSK9 antibody used for immunoprecipitation (anti-native PCSK9 Ab) was raised in rabbits by human *PCSK9 *cDNA vaccination [34]. The anti-PCSK9 antibody used for immunoblotting (anti-IB PCSK9 Ab; (provided by N.G. Seidah) was produced by recombinant PCSK9 vaccination [17]. The anti-LDLR antibody was purchased from Research Diagnostics. Secondary HRP-conjugated antibodies were purchased from Amersham.

### Cell culture and sample collection

HepG2 cells were grown at 37°C in DMEM + 10% FBS + gentamycin 28 μg/mL. For dose response experiments, media was supplemented with a modifier, and cells cultured for an additional 24 hours. Modifiers included simvastatin (0–10 μM; provided by Z. Yao) and fenofibrate (0–200 μM; Sigma). Media was collected in the presence of protease inhibitor cocktail (PIC; Roche). Cells were lysed in 1× RIPA (50 mM Tris-HCl (pH 7.6), 150 mM NaCl, 1% (v/v) NP-40, 0.5% deoxycholate (w/v), 0.1% (w/v) sodium dodecyl sulfate (SDS), PIC) at 4°C for 20 min. Samples were centrifuged at 13,000 × g for 10 min and total cell lysates (TCL) collected. Protein concentrations were determined by BioRad Protein Assay.

### Immunoprecipitation and immunoblotting

Immunoprecipitations (IP) of PCSK9 from cell culture media were carried out following standard protocols with excess anti-native PCSK9 Ab (1:500) and 25 μl of Protein-A agarose (Sigma) overnight at 4°C. Immunoprecipitates and TCL were immunoblotted (IB) following standard protocol. The primary anti-IB PCSK9 was used at 1/1000, the anti-LDLR was used at 1/250 and the secondary antibodies at 1/5000 dilutions. Immunoblots were revealed by chemiluminescence (Western Lightening Plus; Perkin-Elmer) on X-OMAT film (Kodak). The PCSK9 signal was quantified by densitometry using Syngene's Chemigenius 2XE imager and GeneTool software. Experiments were carried out in triplicate.

### Subjects, sample handling and assay procedures

For human studies described below all 59 subjects gave informed written consent and the ethics research committees approved study protocols. The fibrate treatment group included 19 subjects (12 males and 7 females) from a post-hoc study who were recruited from the Ottawa Hospital Lipid Clinic and given fibrate therapy [24]. Subjects were required to have a TC/HDLC ratio ≥ 6.0 for males, 5.6 for females and elevated TGs (2.3–5.5 mmol/L). They were on the American Heart Association/National Cholesterol Education Program Step 1 diet 4–8 weeks prior to receiving fibrates and were not taking lipid-lowering medication. As part of a comparison study for efficacy of fibrates, nine subjects had received 67 mg micronized fenofibrate 3× daily for 24 ± 3 weeks and 10 subjects had received 600 mg gemfibrozil 2× daily for 24 ± 3 weeks, both standard treatment regimes. Blood was collected pre- and post-treatment. Plasma was obtained from blood collected into vacutainer tubes containing EDTA for the fasted fibrate group. TC and TG were measured using enzymatic kits (Boehringer Mannheim) adapted for a Roche Cobas Mira analyzer. HDLC was measured in the supernatant following precipitation of non-HDLC with phosphotungstate on BM/Hitachi 917 analyzer (Roche Diagnostics) and LDLC was calculated as above.

The statin treatment group included forty subjects (31 males and 9 females) from a post-hoc study who were recruited to the Vascular Disease Prevention and Research Centre at the Southeastern Ontario Health Sciences Centre in Kingston (n = 33) or the Ottawa Hospital Lipid Clinic (n = 7). Subjects were required to have elevated LDLC (≥ 3.0 mmol/L). They discontinued all lipid-lowering medication for 4 weeks prior to commencement of atorvastatin to establish baseline levels. Subjects then received a standard 10 mg atorvastatin dose once daily for 6 weeks. Blood was collected pre- and post-treatment. Serum was obtained from blood collected into SST vacutainer tubes for the fasted atorvastatin group. TC, TG and HDLC were measured using Roche test kits on the Roche Modular Instrument. LDLC was calculated by the Friedewald equation.

For post-hoc studies of changes in plasma PCSK9 levels from our statin and fibrate groups the plasma PCSK9 assay was carried out as per Mayne *et al *[17]. All samples were quantified a minimum of 2× with an intra-assay coefficient of variability (CV) of 6.4% and an interassay CV of 7.3%.

### Statistical analysis

All results are expressed as mean ± standard deviation (SD), except where indicated. The differences in lipid parameters between the fenofibrate and gemfibrozil groups were analyzed by Mann-Whitney U test. Pre- and post-treatment data within each treatment group were compared using the Wilcoxon's signed rank test. Spearman correlation coefficients (r) were determined to assess the relationship between different parameters. Data was analyzed using SAS/PC statistical software and GraphPad Prism 5.0 with significance defined as *p *< 0.05.

## Competing interests

The authors declare that they have no competing interests.

## Authors' contributions

JM collected, analysed and compiled study data, had full access to all data in the study, and had final responsibility for preparation and submission of this manuscript for publication, TD carried out cell culture studies and preparation of the manuscript, AR collected and analysed the PCSK9 human data and participated in preparation of the manuscript, MC^2 ^recruited subjects for the Ottawa atorvastatin and fibrate groups, compiled the lipoprotein data and contributed to the manuscript preparation, AC participated in the collection and analysis of the PCSK9 human data, KAL recruited subjects for the Kingston atorvastatin group and compiled the lipoprotein data, SAL supervised the Kingston atorvastatin lipoprotein study and compilation of lipoprotein data, MM co-supervised cell culture studies and manuscript review, TCO supervised the Ottawa atorvastatin and fibrate lipoprotein studies and compilation of lipoprotein data, MC^1 ^participated in data compilation and manuscript review. All authors read and approved the final manuscript.

## References

[B1] Seidah NG, Benjannet S, Wickham L, Marcinkiewicz J, Jasmin SB, Stifani S, Basak A, Prat A, Chretien M (2003). The secretory proprotein convertase neural apoptosis-regulated convertase 1 (NARC-1): liver regeneration and neuronal differentiation. Proc Natl Acad Sci U S A.

[B2] Abifadel M, Rabes JP, Boileau C, Varret M (2007). [After the LDL receptor and apolipoprotein B, autosomal dominant hypercholesterolemia reveals its third protagonist: PCSK9.]. Ann Endocrinol (Paris).

[B3] Abifadel M, Varret M, Rabes JP, Allard D, Ouguerram K, Devillers M, Cruaud C, Benjannet S, Wickham L, Erlich D, Derre A, Villeger L, Farnier M, Beucler I, Bruckert E, Chambaz J, Chanu B, Lecerf JM, Luc G, Moulin P, Weissenbach J, Prat A, Krempf M, Junien C, Seidah NG, Boileau C (2003). Mutations in PCSK9 cause autosomal dominant hypercholesterolemia. Nat Genet.

[B4] Timms KM, Wagner S, Samuels ME, Forbey K, Goldfine H, Jammulapati S, Skolnick MH, Hopkins PN, Hunt SC, Shattuck DM (2004). A mutation in PCSK9 causing autosomal-dominant hypercholesterolemia in a Utah pedigree. Hum Genet.

[B5] Leren TP (2004). Mutations in the PCSK9 gene in Norwegian subjects with autosomal dominant hypercholesterolemia. Clin Genet.

[B6] Humphries SE, Whittall RA, Hubbart CS, Maplebeck S, Cooper JA, Soutar AK, Naoumova R, Thompson GR, Seed M, Durrington PN, Miller JP, Betteridge DJ, Neil HA (2006). Genetic causes of familial hypercholesterolaemia in patients in the UK: relation to plasma lipid levels and coronary heart disease risk. J Med Genet.

[B7] Cohen J, Pertsemlidis A, Kotowski IK, Graham R, Garcia CK, Hobbs HH (2005). Low LDL cholesterol in individuals of African descent resulting from frequent nonsense mutations in PCSK9. Nat Genet.

[B8] Berge KE, Ose L, Leren TP (2006). Missense Mutations in the PCSK9 Gene Are Associated With Hypocholesterolemia and Possibly Increased Response to Statin Therapy. Arterioscler Thromb Vasc Biol.

[B9] Kotowski IK, Pertsemlidis A, Luke A, Cooper RS, Vega GL, Cohen JC, Hobbs HH (2006). A Spectrum of PCSK9 Alleles Contributes to Plasma Levels of Low-Density Lipoprotein Cholesterol. Am J Hum Genet.

[B10] Brown MS, Goldstein JL (2006). Biomedicine. Lowering LDL--not only how low, but how long?. Science.

[B11] Tall AR (2006). Protease variants, LDL, and coronary heart disease. N Engl J Med.

[B12] Horton JD, Shah NA, Warrington JA, Anderson NN, Park SW, Brown MS, Goldstein JL (2003). Combined analysis of oligonucleotide microarray data from transgenic and knockout mice identifies direct SREBP target genes. Proc Natl Acad Sci U S A.

[B13] Maxwell KN, Soccio RE, Duncan EM, Sehayek E, Breslow JL (2003). Novel putative SREBP and LXR target genes identified by microarray analysis in liver of cholesterol-fed mice. J Lipid Res.

[B14] Dubuc G, Chamberland A, Wassef H, Davignon J, Seidah NG, Bernier L, Prat A (2004). Statins upregulate PCSK9, the gene encoding the proprotein convertase neural apoptosis-regulated convertase-1 implicated in familial hypercholesterolemia. Arterioscler Thromb Vasc Biol.

[B15] Alborn WE, Cao G, Careskey HE, Qian YW, Subramaniam DR, Davies J, Conner EM, Konrad RJ (2007). Serum Proprotein Convertase Subtilisin Kexin Type 9 Is Correlated Directly with Serum LDL Cholesterol. Clin Chem.

[B16] Lagace TA, Curtis DE, Garuti R, McNutt MC, Park SW, Prather HB, Anderson NN, Ho YK, Hammer RE, Horton JD (2006). Secreted PCSK9 decreases the number of LDL receptors in hepatocytes and in livers of parabiotic mice. J Clin Invest.

[B17] Mayne J, Raymond A, Chaplin A, Cousins M, Kaefer N, Gyamera-Acheampong C, Seidah NG, Mbikay M, Chretien M, Ooi TC (2007). Plasma PCSK9 levels correlate with cholesterol in men but not in women. Biochem Biophys Res Commun.

[B18] Cunningham D, Danley DE, Geoghegan KF, Griffor MC, Hawkins JL, Subashi TA, Varghese AH, Ammirati MJ, Culp JS, Hoth LR, Mansour MN, McGrath KM, Seddon AP, Shenolikar S, Stutzman-Engwall KJ, Warren LC, Xia D, Qiu X (2007). Structural and biophysical studies of PCSK9 and its mutants linked to familial hypercholesterolemia. Nat Struct Mol Biol.

[B19] Nassoury N, Blasiole DA, Tebon Oler A, Benjannet S, Hamelin J, Poupon V, McPherson PS, Attie AD, Prat A, Seidah NG (2007). The Cellular Trafficking of the Secretory Proprotein Convertase PCSK9 and Its Dependence on the LDLR. Traffic.

[B20] Qian YW, Schmidt RJ, Zhang Y, Chu S, Lin A, Wang H, Wang X, Beyer TP, Bensch WR, Li W, Ehsani ME, Lu D, Konrad RJ, Eacho PI, Moller DE, Karathanasis SK, Cao G (2007). Secreted proprotein convertase subtilisin/kexin-type 9 downregulates low-density lipoprotein receptor through receptor-mediated endocytosis. J Lipid Res.

[B21] Zhang DW, Lagace TA, Garuti R, Zhao Z, McDonald M, Horton JD, Cohen JC, Hobbs HH (2007). Binding of PCSK9 to EGF-A repeat of LDL receptor decreases receptor recycling and increases degradation. J Biol Chem.

[B22] Benjannet S, Rhainds D, Essalmani R, Mayne J, Wickham L, Jin W, Asselin MC, Hamelin J, Varret M, Allard D, Trillard M, Abifadel M, Tebon A, Attie AD, Rader DJ, Boileau C, Brissette L, Chretien M, Prat A, Seidah NG (2004). NARC-1/PCSK9 and its natural mutants: zymogen cleavage and effects on the low density lipoprotein (LDL) receptor and LDL cholesterol. J Biol Chem.

[B23] Staels B, Fruchart JC (2005). Therapeutic roles of peroxisome proliferator-activated receptor agonists. Diabetes.

[B24] Dzavik V AJC (1999). Micronised fenifibrate or gemfibrozil in patients with combined hyperlipidemia: a double-blind randomized multicentre trial. Atherosclerosis.

[B25] Tobert JA (2003). Lovastatin and beyond: the history of the HMG-CoA reductase inhibitors. Nat Rev Drug Discov.

[B26] Staels B, Dallongeville J, Auwerx J, Schoonjans K, Leitersdorf E, Fruchart JC (1998). Mechanism of action of fibrates on lipid and lipoprotein metabolism. Circulation.

[B27] Kourimate S, Le May C, Langhi C, Jarnoux AL, Ouguerram K, Zair Y, Nguyen P, Krempf M, Cariou B, Costet P (2008). Dual mechanisms for the fibrate-mediated repression of proprotein convertase subtilisin/kexin type 9. J Biol Chem.

[B28] Rouini MR (2006). Study of Dose-linearity of gemfibrozil Pharmokinetics in Humans. International Journal of Pharmacology.

[B29] Harvengt C, Desager JP (1980). Lack of pharmacokinetic interaction of colestipol and fenofibrate in volunteers. Eur J Clin Pharmacol.

[B30] Amemiya-Kudo M, Shimano H, Hasty AH, Yahagi N, Yoshikawa T, Matsuzaka T, Okazaki H, Tamura Y, Iizuka Y, Ohashi K, Osuga J, Harada K, Gotoda T, Sato R, Kimura S, Ishibashi S, Yamada N (2002). Transcriptional activities of nuclear SREBP-1a, -1c, and -2 to different target promoters of lipogenic and cholesterogenic genes. J Lipid Res.

[B31] Grozdanov PN, Petkov PM, Karagyozov LK, Dabeva MD (2006). Expression and localization of PCSK9 in rat hepatic cells. Biochem Cell Biol.

[B32] Costet P, Cariou B, Lambert G, Lalanne F, Lardeux B, Jarnoux AL, Grefhorst A, Staels B, Krempf M (2006). Hepatic PCSK9 expression is regulated by nutritional status via insulin and sterol regulatory element-binding protein 1c. J Biol Chem.

[B33] Careskey HE, Davis RA, Alborn WE, Troutt JS, Cao G, Konrad RJ (2008). Atorvastatin increases human serum levels of proprotein convertase subtilisin/kexin type 9. J Lipid Res.

[B34] Chowdhury PS, Gallo M, Pastan I (2001). Generation of high titer antisera in rabbits by DNA immunization. J Immunol Methods.

